# A smartphone application for semi-automated QT interval analysis based on a snapshot of an electrocardiogram trace displayed on a patient monitor

**DOI:** 10.1007/s10877-025-01277-z

**Published:** 2025-04-10

**Authors:** David Beckmann, Moritz Flick, Karim Kouz, Bernd Saugel

**Affiliations:** 1 Department of Anesthesiology, Center of Anesthesiology and Intensive Care Medicine, University Medical Center Hamburg-Eppendorf, Martinistrasse 52, 20246 Hamburg, Germany; 2 Outcomes Research Consortium, Cleveland, OH USA

**Keywords:** Anesthesia, ECG, Hemodynamic monitoring, QT interval, QTc interval

## Abstract

We developed a smartphone application (SMART-QT application) that can semi-automatically measure QT and QTc intervals based on a snapshot of the electrocardiogram (ECG) trace and the heart rate displayed on a patient monitor. In this study, we aimed to validate the SMART-QT application. In this prospective single-center method comparison study, we measured QT and QTc intervals with the SMART-QT application (QT_APP_ and QTc_APP_; test method) and simultaneously manually measured QT and QTc intervals from a 12-lead ECG (QT_REF_ and QTc_REF_; reference method) in 57 adult volunteers and patients who had sinus rhythm and no acute or chronic cardiac comorbidities. To investigate the agreement between QT_APP_ and QT_REF_ and between QTc_APP_ and QTc_REF_, we performed Bland-Altman analyses and calculated the mean of the differences, the standard deviation, and the 95%-limits of agreement (95%-LOA). We defined clinically acceptable agreement as maximum mean of the differences ± standard deviation of 20 ± 20 ms. The mean of the differences between QT_APP_ and QT_REF_ was 14 ± 20 ms (95%-LOA −26 to 54 ms). The mean of the differences between QTc_APP_ and QTc_REF_ was 13 ± 15 ms (95%-LOA −16 to 42 ms). The agreement between QT_APP_ and QT_REF_ and between QTc_APP_ and QTc_REF_ was clinically acceptable in adult volunteers and patients without cardiac comorbidities.

**Supplementary Information:**

The online version contains supplementary material available at 10.1007/s10877-025-01277-z.

## Background

Continuous electrocardiogram (ECG) monitoring is mandatory in all patients having surgery with general anesthesia [1–3]. Perioperative ECG monitoring is performed using a 3-lead or a 5-lead ECG – with the ECG trace being continuously displayed on the patient monitor.

On the ECG, the QT interval represents the time from cardiac depolarization to repolarization of the ventricles. The QT interval depends on heart rate. To make QT intervals at different heart rates comparable, the QT interval can be corrected for heart rate – i.e., normalized to a heart rate of 60 beats/min – resulting in the QTc interval [4].

A prolongation of the QT interval – or long QT syndrome – is associated with an increased risk for cardiac arrhythmias. Long QT syndrome can be inherited or caused by drugs. Many drugs frequently used in perioperative medicine – such as anesthetics, antibiotics, antiemetics, and antipsychotics [5]– can prolong the QT interval.

Patients under general anesthesia are at risk of developing QT prolongation – especially when they need to be given drugs that prolong the QT interval [5]. A prolonged QT interval can be found in half of the patients under general anesthesia [6]. However, QT intervals are not routinely monitored in patients having surgery.

We, therefore, developed a smartphone application (SMART-QT application) that can semi-automatically measure QT and QTc intervals based on a snapshot of the ECG trace and the heart rate displayed on a patient monitor. The aim of this study was to investigate the agreement between QT and QTc intervals measured with the SMART-QT application (QT_APP_ and QTc_APP_; test method) and QT and QTc intervals manually measured from a 12-lead ECG (QT_REF_ and QTc_REF_; reference method).

## Methods

### Study design and subjects

This prospective single-center method comparison study was performed between December 2021 and April 2022 in the Department of Anesthesiology, Center of Anesthesiology and Intensive Care Medicine, University Medical Center Hamburg-Eppendorf, Hamburg, Germany. The study protocol was approved by the ethics committee (Ethikkommission der Ärztekammer Hamburg, Hamburg, Germany; Chairperson: Prof. R. Stahl; ethics committee number PV4701; operation number 2021-100705-BO-ff). We included adult volunteers and patients who had sinus rhythm, who had no acute or chronic cardiac comorbidities, who were not taking drugs potentially affecting the QT interval, and who were not pregnant. Written informed consent was obtained from all subjects.

#### SMART-QT application and hardware

The SMART-QT application allows semi-automated measurement of QT and QTc intervals based on a snapshot of the ECG trace and the heart rate displayed on a patient monitor. The SMART-QT application is a progressive web application – a software application delivered through the web – with a server and a client component. For this study, the server component was placed on a small single-board computer (Rasperry Pi; Version 4, Model B, 4 GB). We connected the computer with a lightning-to-ethernet cable to a smartphone (Apple iPhone SE; Apple, Cupertino, CA, USA) with the computer acting as access point.

To measure QT and QTc intervals with the SMART-QT application the user takes a snapshot of an ECG trace displayed on a patient monitor. The snapshot appears on the smartphone display and three sliders are manually placed to mark two neighboring Q waves and the T wave in-between. The current version of the application requires manual input of heart rate. The SMART-QT application then computes QT_APP_ and QTc_APP_ using the Bazetts formula as: QTc_APP_ = QT_APP_/√(RR/1.000) [7].

#### Study measurements

All measurements were performed while the subjects were resting in supine position. We simultaneously recorded a 12-lead ECG with a writing speed of 25 mm/sec (40 ms/mm) using the ECG recorder Cardiovit AT-102 plus (Schiller, Baar, Switzerland) and a 3-lead ECG using the patient monitor Infinity Delta (Draeger, Lübeck, Germany).

We manually measured the reference QT interval from the 12-lead ECG paper printout (QT_REF_). Additionally, we manually measured the RR-interval from the 12-lead ECG paper printout, calculated the heart rate as 60.000/RR, and calculated the reference QTc interval (QTc_REF_) using the Bazetts formula.

While recording the 12-lead ECG, we simultaneously took a snapshot of the ECG trace and the heart rate displayed on the patient monitor and measured the QT interval and QTc interval with the SMART-QT application (QT_APP_ and QTc_APP_) (test method).

We additionally took a snapshot of the ECG paper printout and the HR calculated from the ECG paper printout and measured the QT interval and the QTc interval with the SMART-QT application (QT_APP−PAPER_ and QTc_APP−PAPER_).

By inducing an artifact in the ECG, we ensured that the exact same QRS complexes from the 12-lead ECG and the ECG lead from the patient monitor were analyzed. Lead II was used for all measurements.

#### Statistical analysis

Descriptive statistics are presented as median with interquartile range for continuous data and as absolute frequency and percentage for categorical data.

We use scatter plots to illustrate the relationship between QT_APP_ and QT_REF_ and QTc_APP_ and QTc_REF_. To investigate the agreement between QT_APP_ and QT_REF_ and between QTc_APP_ and QTc_REF_, we performed Bland-Altman analyses and calculated the mean of the differences (reference minus test method; e.g. QT_REF_ minus QT_APP_ and QTc_REF_ minus QTc_APP_), the standard deviation (SD) of the differences, and the 95%-limits of agreement (95%-LOA; mean of the differences ± 1.96 × SD) with corresponding 95%-confidence intervals (95%-CI). We defined clinically acceptable agreement as maximum mean of the differences ± SD of 20 ± 20 ms [8]. Additionally, we assessed the number of measurement pairs with excellent agreement (< 20 ms difference), acceptable agreement (< 40 ms difference), and unacceptable agreement (> 40 ms difference) [9]. We additionally analyzed Pearson’s correlation coefficient. The same analyses were performed to compare QT_APP−PAPER_ and QTc_APP−PAPER_ with QT_REF_ and QTc_REF_.

We used Medcalc version 22.030 (MedCalc Software Ltd; Ostend, Belgium) for statistical analysis.

## Results

We included 60 subjects, but needed to excluded three – two because the 12-lead ECG paper printout could not be synchronized with the ECG trace on the patient monitor and one because the 12-lead ECG could not properly be recorded. We thus analyzed data of 57 subjects with a total of 57 measurement pairs (Table 1).

**Table 1:** Subject characteristics

Age, years	51 ± 16
Sex, female	25 (44%)
Height, cm	174 ± 9
Body weight, kg	79 ± 19
BMI, kg/m^2^	26 ± 5
ASA physical status class, (I/II/III/IV)	17 (30%)/ 20 (35%)/ 16 (28%)/ 4 (7%)

Data are presented as mean ± standard deviation or absolute number (percentage)

**Fig. 1:**
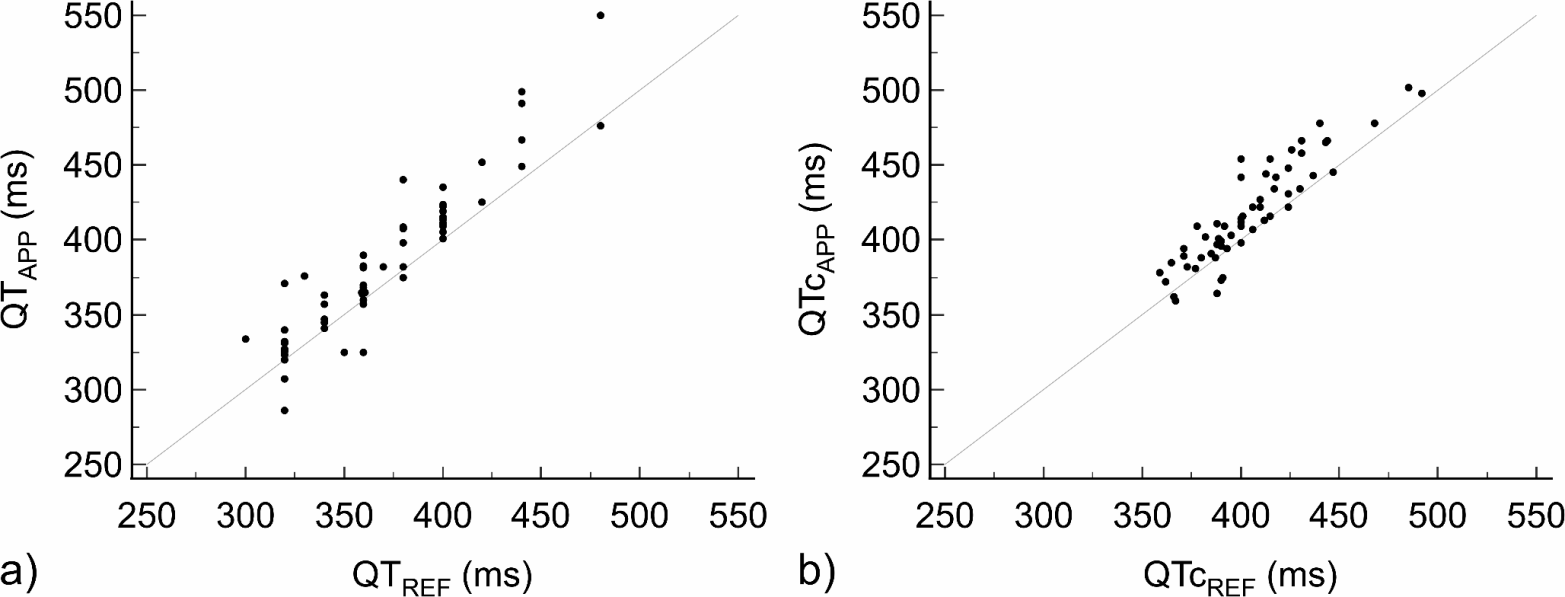
Scatter plot illustrating the relation between QT and QTc intervals measured with the SMART-QT application from the patient monitor (QT_APP_ and QTc_APP_; test method) and manually measured QT and QTc intervals from a 12-lead ECG (QT_REF_ and QTc_REF_; reference method)

**Fig. 2:**
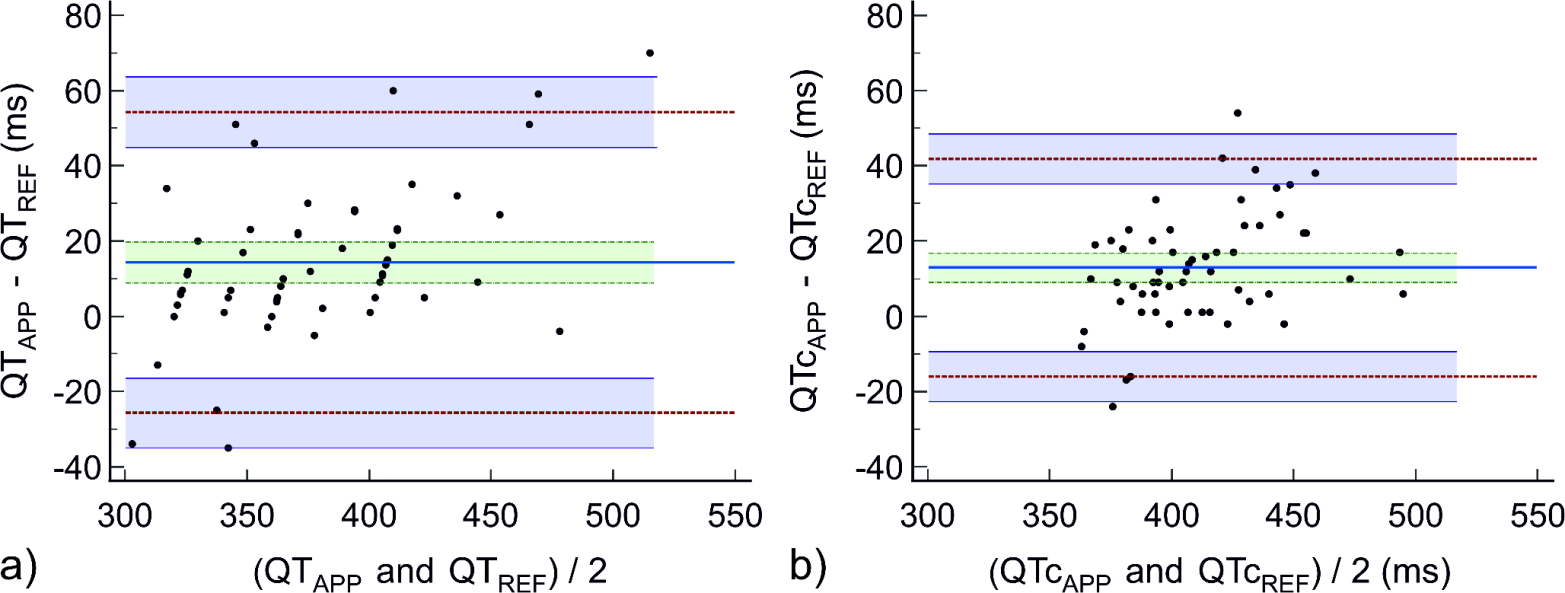
Bland-Altman plot illustrating mean of the differences (bold horizontal line) and 95% limits of agreement (upper and lower dotted horizontal lines) between QT and QTc intervals measured with the SMART-QT application from the patient monitor (QT_APP_ and QTc_APP_; test method) and manually measured QT and QTc intervals from a 12-lead ECG (QT_REF_ and QTc_REF_; reference method). Shaded areas represent 95%- CI

The mean ± SD QT_APP_ was 371 ± 42 ms and the mean QT_REF_ was 386 ± 52 ms with a correlation coefficient of 0.93 (*P* < 0.001) (Fig. 1a). The mean of the differences between QT_APP_ and QT_REF_ was 14 ± 20 ms (95%-LOA of -26 to 54 ms) (Fig. 2a; Table 2). 61% of the measurement pairs had excellent, 28% acceptable, and 11% unacceptable agreement.

**Table 2:** Results

	QT_APP_ vs. QT_REF_	QTc_APP_ vs. QTc_REF_
Mean of the differences (95%-CI), ms	14 (9 to 20)	13 (9 to 17)
Standard deviation of the mean of the differences, ms	20	15
Lower limit of agreement (95%-CI), ms	-26 (-35 to -16)	-16 (-23 to -9)
Upper limit of agreement (95%-CI), ms	54 (45 to 64)	42 (35 to 49)

Data are presented as mean ± standard deviation or absolute number (percentage); 95%-CI – 95%-confidence interval

The mean QTc_APP_ was 405 ± 29 ms and the mean QTc_REF_ was 418 ± 34 ms with a correlation coefficient of 0.91 (*P* < 0.001) (Fig. 1b). The mean of the differences between QTc_APP_ and QTc_REF_ was 13 ± 15 ms (95%-LOA of -16 to 42 ms) (Fig. 2b; Table 2). 70% of the measurement pairs had excellent, 26% acceptable, and 4% unacceptable agreement.

The mean QT_APP−PAPER_ was 374 ± 42 ms and the mean QTc_APP−PAPER_ was 408 ± 29 ms. The correlation coefficient between QT_APP−PAPER_ and QT_REF_ was 0.99 (*P* < 0.001) and between QTc_APP−PAPER_ and QTc_REF_ 0.97 (*P* < 0.001) (Supplementary Fig. 1). The mean of the differences between QT_APP−PAPER_ and QT_REF_ was 3 ± 6 ms (95%-LOA of -8 to 14 ms) and between QTc_APP−PAPER_ and QTc_REF_ 3 ± 7 ms (95%-LOA of -10 to 16 ms) (Supplementary Fig. 2; Supplementary Table 1). All QT_APP−PAPER_ and QTc_APP−PAPER_ measurements had excellent agreement with their respective reference.

## Discussion

In this prospective method comparison study, the agreement between QT_APP_ and QT_REF_ and between QTc_APP_ and QTc_REF_ was clinically acceptable. About 90% of the QT_APP_ and 95% of QTc_APP_ measurements had excellent (< 20 ms) or acceptable (< 40 ms) agreement with their reference measurements.

The SMART-QT application allows measuring QT and QTc intervals based on a snapshot of an ECG trace displayed on a patient monitor – and thus only requires a smartphone with a camera. The SMART-QT application could thus be used to measure QT and QTc intervals in all patients in whom an ECG trace is displayed on a monitor – e.g., patients treated in emergency departments, operating rooms, or high dependency and intensive care units. In contrast to the SMART-QT application, other applications or smart devices – such as the KardiaMobile (AliveCor, MountainView, CA, USA) or the apple watch (Apple, Cupertino, CA, USA) [10] – often require external ECG input [11] or come with sensors for single-lead ECG recording.

The SMART-QT application is supposed to be primarily used for bedside analysis of snapshots of ECG traces displayed on patient monitors. However, it can also be used to measure QT and QTc intervals based on a snapshot of an ECG paper printout instead of the ECG trace displayed on the patient monitor. We thus also measured QT_APP−PAPER_ and QTc_APP−PAPER_. The agreement between QT_APP−PAPER_ and QT_REF_ and between QTc_APP−PAPER_ and QTc_REF_ was excellent.

QT and QTc interval analysis with the SMART-QT application is semi-automated and requires manually marking the Q and T waves on the snapshot. Fully automated detection of the Q and T waves would be possible and would make the measurement operator independent. However, manual detection of the beginning of the Q wave and ending of the T wave presumably would be less accurate than automated detection – particularly because detecting the end of the T wave is challenging [12].

We meticulously ensured that we analyzed the same cardiac cycles manually on the ECG print out and with the SMART-QT application. However, we did not average QT and QTc interval measurements over 3–5 cardiac cycles as recommended [13]. Also, we did not address potential observer variability in reference QT and QTc interval measurements as all measurements were performed by a single investigator. A further limitation is that this was a single center study in volunteers and patients without cardiac comorbidities. Therefore, all patients had a normal QT interval. Our results may thus not be generalizable and further research is required to determine the measurement performance of the SMART-QT application in patients with cardiac comorbidities – especially those that cause ECG alterations.

## Conclusion

The agreement between QT_APP_ and QT_REF_ and between QTc_APP_ and QTc_REF_ was clinically acceptable in adult volunteers and patients without cardiac comorbidities. Pending further validation, the SMART-QT application could be used to measure QT and QTc intervals at the bedside based on a snapshot of an ECG trace displayed on a patient monitor.

## Electronic supplementary material

Below is the link to the electronic supplementary material.

Supplementary Material 1

## Data Availability

No datasets were generated or analysed during the current study.
