# Supplementary material for: A smartphone application for semi-automated QT interval analysis based on a snapshot of an electrocardiogram trace displayed on a patient monitor
Source: J Clin Monit Comput. 2025 Apr 10;39(4):787–91. doi: 10.1007/s10877-025-01277-z (PMC12304064; doi:10.1007/s10877-025-01277-z)
Supplement: Supplementary file 1 — Supplementary Material 1 [file 10877_2025_1277_MOESM1_ESM.pdf]

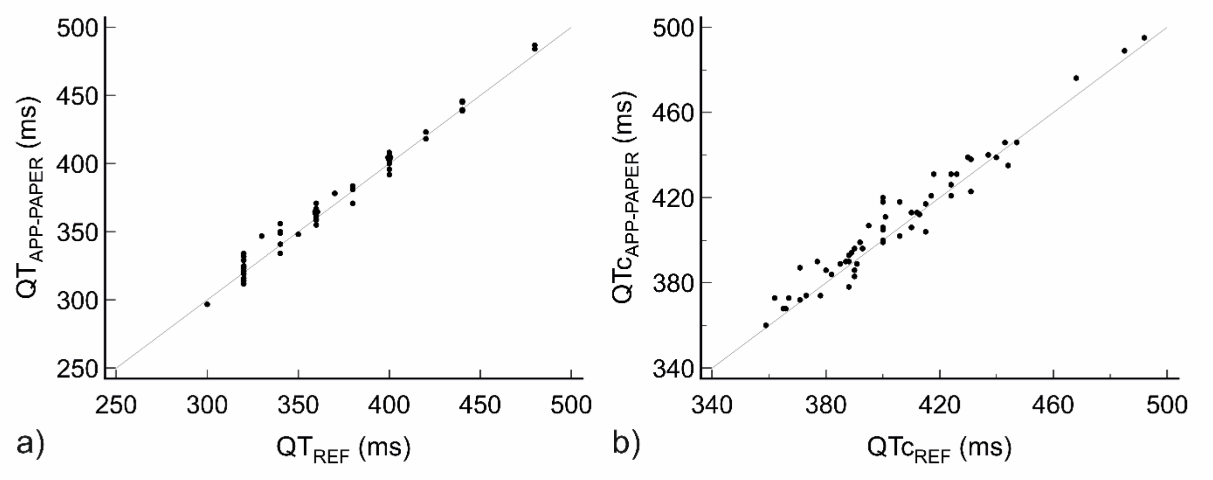

Figure S1: Scatter plot illustrating the relation between QT and QTc intervals measured with the SMART-QT application from the ECG paper printout ($QT_{APP-PAPER}$ and $QTc_{APP-PAPER}$; test method) and manually determined QT and QTc intervals from a 12-lead ECG (QT_{REF} and QTc_{REF}; reference method).

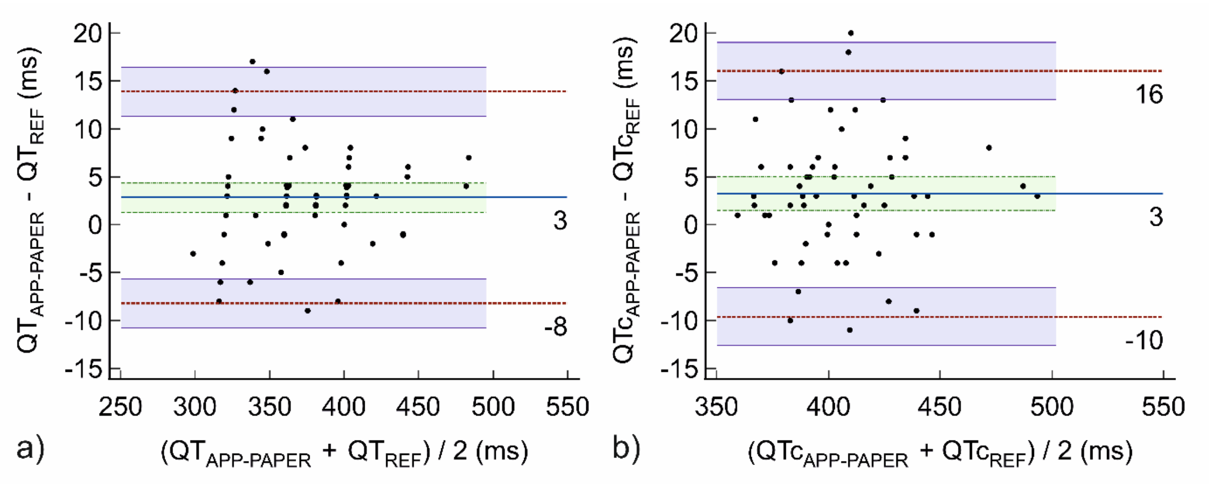

Figure S2: Bland-Altman plot illustrating mean of the differences (bold horizontal line) and 95% limits of agreement (upper and lower dotted horizontal lines) between QT and QTc intervals measured with the SMART-QT application from the ECG paper printout ($QT_{APP-PAPER}$ and $QTc_{APP-PAPER}$; test method) and manually determined QT and QTc intervals from a 12-lead ECG (QT_{REF} and QTc_{REF}; reference method). Shaded areas represent 95% CI.

Table S1

	$QT_{APP\text{-}PAPER}$ vs. QT_{REF}	$QTc_{APP\text{-}PAPER}$ vs. QTc_{REF}
Mean of the differences (95%-CI), ms	3 (1 to 4)	3 (1 to 5)
Standard deviation of the mean of the differences, ms	6	7
Lower limit of agreement (95%-CI), ms	-8 (-11 to -6)	-10 (-13 to -7)
Upper limit of agreement (95%-CI), ms	14 (11 to 16)	16 (13 to 19)

95%-CI – 95%-confidence interval
